# Wettability and Adhesion of Polyethylene Powder Treated with Non-Equilibrium Various Gaseous Plasma in Semi-Industrial Equipment

**DOI:** 10.3390/ma15020686

**Published:** 2022-01-17

**Authors:** Hana Jelínek Šourková, Zuzana Weberová, Jakub Antoň, Petr Špatenka

**Affiliations:** Department of Materials Engineering, Faculty of Mechanical Engineering, Czech Technical University in Prague, Karlovo Náměstí 13, 121 35 Prague, Czech Republic; hana.sourkova@fs.cvut.cz (H.J.Š.); zuzana.weberova@fs.cvut.cz (Z.W.); jakub.anton@fs.cvut.cz (J.A.)

**Keywords:** polyethylene (LLDPE) powder, plasma treatment, wettability, adhesion, uniformity

## Abstract

Plasma treatment of polyethylene powder was carried out in low-pressure gaseous plasma sustained in a semi-industrial reactor powered with a microwave source, in which it was specifically worked with the residual atmosphere. Timed applications of plasma-treated powder in air atmosphere were carried out to study their influence on the adhesion. Based on wettability and adhesion, a treatment time of 5 min was selected for the study of other working gases (nitrogen, oxygen, hydrogen, argon and a mixture of nitrogen and hydrogen). The measurements of wettability showed the highest adhesion increase for nitrogen. The highest increase of adhesion and of surface oxygen contain shown by oxygen treatment. By contrast, treatment with hydrogen resulted in increased roughness of the sintered surface of the powder. The selection of appropriate working gases which are not standard in industrial processes enables one to atypically regulate the adhesion or wettability.

## 1. Introduction

The surface properties of different materials are often inadequate, and must be modified to achieve the desired surface finish [1]. Many polymers are hydrophobic and do not exhibit the wettability needed for specific applications. Different techniques can be used for surface modification, but in recent decades, plasma treatment has become the standard method. Polymers and polymer composites are exposed to non-equilibrium gaseous plasma to interact with particles that are likely to be present in this state of the gas. The non-equilibrium gaseous plasma is a source of free electrons and positively charged ions, as well as chemically reactive radicals formed upon dissociation and/or excitation of the source-gas molecules. The radicals include neutral atoms in their ground and excited states with a reasonable lifetime, such as metastable atoms, for which relaxation by electrical dipole radiation is forbidden by the laws of quantum mechanics, other molecular fragments in metastable states of high excitation energy, and newly formed molecules due to interactions between various particles such as ozone. Negatively charged ions might also be present in gaseous plasma, especially in plasma sustained in electro-negative gases. Furthermore, gaseous plasma glows, so it is a source of radiation in the visible range of wavelengths. The radiation also appears in the invisible range; the most important type of radiation for the surface modification of solid materials is radiation in the ultraviolet (UV) and vacuum ultraviolet (VUV) range of wavelengths.

A polymer sample exposed to gaseous plasma is therefore subjected to a flux of electrons, positively charged ions, neutral atoms and molecules in the ground and excited states, and radiation. The fluxes depend on the densities and concentrations of different gases present in the processing chamber, the type of discharge used for plasma generation, the discharge-to-plasma coupling, the discharge power and power density (i.e., the power per volume or surface, depending on the configuration) and the peculiarities of the samples exposed to plasma. Plasma treatment is regarded as a technique for the modification of surfaces without affecting the bulk properties of the treated materials. Since the radiation usually penetrates rather deeply into a polymer, the sub-surface film is often modified as well. For example, the VUV radiation arising from oxygen or hydrogen plasma causes significant modifications of polymers, as shown recently by Hori’s group [2]. Even atoms may diffuse rather deeply into the polymer structure and penetrate up to 20–40 nm into the low-density polyethylene upon continuous treatment with gaseous plasma, as shown by Reniers’ group [3]. Nevertheless, most of the effects of plasma treatment are limited to the surface layer, with a thickness of a few nanometers. Different techniques are used to investigate these modifications, and the most popular one measures the liquid contact angle (to determine wettability) [4]. The modifications of surface composition and structure are often determined by means of X-ray photoelectron spectroscopy (XPS) [5], whereas any changes in topography can be determined by scanning electron microscopy (SEM) and atomic force microscopy (AFM) [6].

Polyethylene (PE) has been treated by gaseous plasma for decades. Lehocky et al. [7] reported the RF-plasma treatment of HDPE as an effective tool for improving wettability, as well as for increasing its surface micro-hardness. They found a significant decrease in both advancing and receding contact angles for different liquids after a minute of plasma treatment. Abou et al. [8] used a linear torch to sustain the plasma in a mixture of helium and oxygen and reported a final static water contact angle (WCA) of 31°. Experiments with a showerhead plasma torch enabled a static WCA of 37°. The oxygen-to-carbon ratio (O/C) after the plasma treatment, as determined by XPS, was 0.36 in both cases. Lopez-Santos et al. [9] used remote microwave (MW) plasma sustained in a mixture of argon and oxygen and found a static WCA of 30°, but the O/C as determined by XPS was only 0.2. The same group also reported experiments with plasma sustained by dielectric barrier discharge (DBD). The static WCA was 34°, but the O/C as large as 0.55 [9]. A simple direct current (DC) glow discharge was used for the plasma treatment of polyethylene for as long as 10 min by Pandiyaraj et al. [10]. They used plasma in different gases and reported the static WCA as low as 25° when the case plasma was sustained in a mixture of argon and oxygen. Pure oxygen plasma treatment resulted in a WCA of 35°. Treatment in the same conditions but in air plasma enabled the static WCA of 32°. The reported O/C ratios for these samples did not follow the behavior of WCA. The O/C were 0.18, 0.11, and 0.22, respectively. One of the largest O/C ratios (as much as 0.68) was reported by Borcia et al. [11], who used DBD plasma in air. In spite of the extremely high oxygen concentration, as determined by XPS, the reported static WCA remained as high as 54°. The brief survey in this paragraph indicates a non-trivial correlation between the oxygen concentration at the surface film as probed by XPS and the wettability as probed by WCA. A more thorough review of such correlations recently appeared in [12].

Even more challenging is plasma activation of polyethylene in the form of powder. When the particle size of powder is below the order of millimeters, it is not feasible to measure the water contact angle. Instead, different techniques are applicable for the determination of the surface wettability. The Washburn method, which was reported a century ago [13], is commonly used for this purpose. This technique was used by Patra et al. [14], who observed the improved wettability of PE powder after treatment with a low-pressure plasma sustained in different gases. Both air and oxygen plasma treatments increased the wettability by approximately two times, compared with nontreated PE powder. A slightly smaller increase was observed after treatment in a mixture of oxygen and hydrogen. A recent review on the plasma treatment of polymer powders was published in [15]. That comprehensive paper describes both laboratory research and industrial applications. Sonnenfeld et al. [16] used a capacitively coupled radio-frequency (RF) plasma reactor to improve the wettability and flowability of fine powders made from high-density PE. The formation of floating potential sheaths around each single particle immersed in the plasma enabled intense particle/plasma interaction and thus improved wettability in a treatment time of only 0.12 s. The O/C ratio after this treatment was 0.15. In another paper, the same group reported XPS results which indicated the formation of CO and COOH functional groups on the powder surface [17]. Park and Kim [18] treated high-density PE powder in a fluidized bed reactor with inductively coupled RF plasma and found a linear relationship between the surface wettability and the O/C concentration, which was as high as 0.35 under the optimal treatment conditions. A similar reactor was also found to be useful by Bretagnol et al. [19], who treated low-density PE in nitrogen plasma. They found a gradual increase in the O/C ration with plasma treatment time and observed O/C = 0.13 after about 2 min. The surface was also well-functionalized with nitrogen functional groups, as the final N/C ratio was 0.11.

The cited literature reports different surface finishes, which can be explained by the peculiarities of plasma reactors. The influence of plasma sustained in different gases on the surface finish is thus difficult to deduce. This is especially a problem for setting up the process in the industry, where it is difficult to achieve a system with minimal leakage. In industrial applications, XPS is extremely expensive and lengthy and cannot be used by default. For this reason, industry mostly relies on faster and cheaper methods, based on wettability changes. Typically, wettability correlates with the binding of polar function groups to the surface (oxygen), as described in detail above. However, in the case of the plasma treatment of powders in different gases on an industrial or semi-industrial scale, the change in wettability may not correspond to a change in surface chemistry. In order to get an insight into the surface finish of polyethylene, we used plasma sustained in the same semi-industrial reactor with the same discharge conditions but in different gases, which are standard in industry use. Together with investigating the change in wettability and adhesion, it can bring new insights into the principle of plasma, affecting the structurally very complex and large surface that polymer powders have.

Plasma-treated powders are constantly finding new applications, and their development is starting to concern the industrial sphere [15], where they can be used as fillers in water-based paint due to their increased wettability, without the use of detergents. For composite materials, the formation of adhesion between the filler and matrix is the critical parameter. Here adhesion can be mediated precisely by the creation of new functional groups that can promote better wettability (mechanical anchoring) or create a chemical bond [20]. Therefore, this study focuses on the influence of wettability adhesion and the number of function groups on plasma-treated powder surfaces, depending on plasma treatment parameters.

## 2. Materials and Methods

Commercially available PE powder with an average particle size of 160 µm was supplied by Dow corporate (Midland, MI, USA). The product’s name is Dowlex™ 2629.10UE Polyethylene Resin. It is a hexane-based linear low-density polyethylene (LLDPE) with a melt flow index of 3.8 g/min (ASTM D1238), a density of 0.9370 g/cm^3^ (ASTM D792), and a bulk density of 0.346 g/cm^3^ (ASTM D1895).

**Figure 1:**
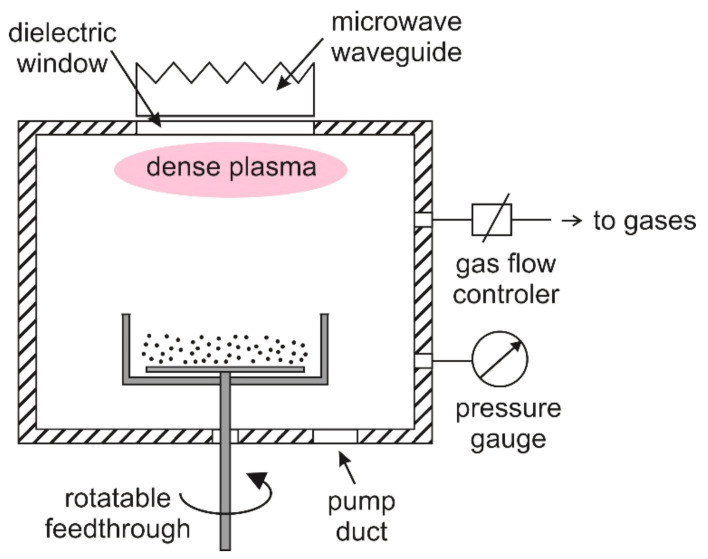
Schematic of the experimental setup for the treatment of PE powder.

The powder was treated in a LA400 semi-industrial reactor produced by Surface Treat Ltd. (Surface Treat Ltd., Turnov, Czech Republic), described in more detail in [21]. The discharge chamber is a cubicle of dimensions 4 × 4 × 4 dm^3^. The upper flange of the discharge chamber is equipped with a microwave source. The source enables the sustenance of microwave plasma in the upper part of the discharge chamber, as shown schematically in Figure 1. The rest of the discharge chamber was occupied by a diffusing plasma of roughly uniform brightness at the pressure selected for these experiments, i.e., 100 Pa. The discharge chamber was pumped with a two-stage rotary pump of a nominal pumping speed, 65 m^3^/h. There was a butterfly valve (not shown in Figure 1) between the discharge chamber and the vacuum pump, which enabled the adjustment of the gas pressure inside the discharge chamber independently from the gas flow. Gases were introduced into the discharge chamber through gas-flow controllers calibrated for different gases. For the experiments reported in this paper, we used ambient air, nitrogen, oxygen, hydrogen, argon and a mixture of nitrogen and hydrogen (50:50).

A stainless-steel dish was placed in the center of the discharge chamber, as shown in Figure 1. There was a stirring device inside the dish; the device was on rotatable feedthrough, as shown in Figure 1. The rotation speed was set to 40 rpm. The dish was filled with the polymer powder, the discharge chamber was closed, the pump was turned on for a minute and the selected gas was introduced by continuous pumping. The butterfly valve was adjusted so that the pressure inside the discharge chamber was 100 Pa.

A quantity of 250 g of polymer powder was placed into the stainless-steel dish as explained above and the experimental system was pumped with the rotary pump. The pressure stabilized at about 12 Pa after pumping for three minutes, and the contribution of the residual atmosphere was calculated at 10 sccm. Ambient air leaked into the discharge chamber at the flow rate of 100 sccm upon continuous pumping, and the butterfly valve was adjusted so that a constant pressure of 100 Pa was established. The stirring device and the MW generator were turned on, and the powder was treated for a period of one minute. The discharge chamber was vented after accomplishing the plasma treatment and the powder was probed for wettability, surface functionalization and shear strength on metal. The experiments with air were repeated for other treatment times, i.e., 5, 10 and 20 min.

The discharge chamber was vented after the treatment, and the powder was characterized using the Washburn method and XPS. The wettability of the powder material was measured using self-constructed equipment and determined with a tensiometer from dynamic capillarity rising measurements according to the Washburn method [13]. Benzyl alcohol was used at 25 °C as a liquid for measuring the penetration in the powder in the specific testing conditions. Values of suction (g^2^/s) were normalized to the value measured for nontreated powder. The wettability was expressed as a percentage of increased suction, with 100% assigned to nontreated powder. The measurements of wettability were repeated several times for each sample.

The concentration of oxygen on the plasma-treated powder was measured by means of X-ray photoelectron spectroscopy (XPS) (Thermo VG Scientific, East Grinstead, UK). The measurement was performed under the pressure of residual gases in the analyzer chamber of a spectrometer of 5 × 10^−10^ mbar. Monochromatic radiation A1 Kα (hʋ = 1486.6 eV) was used for the excitation of photoelectrons. Spectra in the range of binding energies of 0–1000 eV were measured together with the measurement of difference line C 1s and O 1s in high-resolution mode. The measurement error of the difference in the binding energies was about ± 0.2 eV. The composition of the surface film, as probed by XPS, was determined from the survey spectra, and concentration of various functional groups from the high-resolution C1s spectra. Software XPSPEAK 4.1. (Wise Solutions, Plymouth, MI, USA) was used for fitting of the Gauss–Lorentzian functions.

Adhesion was characterized by shear strength, measured using a modified pin-collar test, a method adapted for the measurement of the adhesion of polymer powders to solid substrates. The same procedure as that described by Weberova et al. [22] was used. Rods made from chrome-plated steel C35 with a fine smooth surface (Ra_max_ = 0.2 µm, average Ra = 0.13 µm) with d = 12 mm, l = 45 mm (Hydraulics s.r.o., Slopné, Czech Republic), were used as a metal substrate. Samples were prepared by coating heated rods with a layer of the investigated powder. The powder was then sintered via further exposure to heat in a furnace to obtain a uniform plastic layer. Coated rods were placed in special molds, which were then filled with more powder and placed in a furnace for final sintering of the plastic mass. To measure the shear strength, rods were pushed out from the plastic collars in the axial direction.

Surface roughness was determined by a Hommel Tester T1000 profilometer (Jenoptik, Jena, Germany). Measurement was carried out on the sintered surface of polymer powder on the metal rod with the following parameters: whole length 4.8 mm, L_c_ = 0.8 mm, v_t_ = 0.5 mm/s, with filter M1 (DIN EN ISO 11562-1) and Rz (µm) was determined. Pictures of the profile of a metal rod with the sintered powder was taken using a DSX1000 digital microscope (Olympus corp., Tokyo, Japan).

## 3. Results

**Figure 2:**
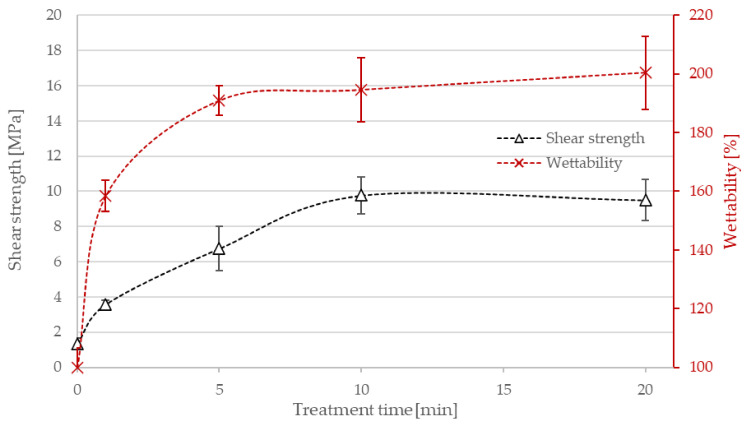
The wettability, shear strength and chemical bond surface concentration of PE powder versus the treatment time using air plasma.

The plasma treatment of polyethylene powder was first performed in an air atmosphere. Figure 2 shows the changes in wettability and adhesion with treatment time in this atmosphere. Even a 1-min treatment increased wettability up to about 160% and shear strength from 1.3 MPa (nontreated powder) to 3.6 MPa. As mentioned above, the wettability of nontreated powder was assigned as 100%. The 5-min treatment further increased the wettability to 190% and the shear strength to 6.7 MPa. Thereafter, the wettability changes were rather marginal, but adhesion increased to 9.8 MPa for the 10 min treatment. Then, no other major changes were seen, as revealed in Figure 2 and the summarized results therefore indicate almost complete surface wettability and adhesion after the treatment with air plasma.

**Figure 3:**
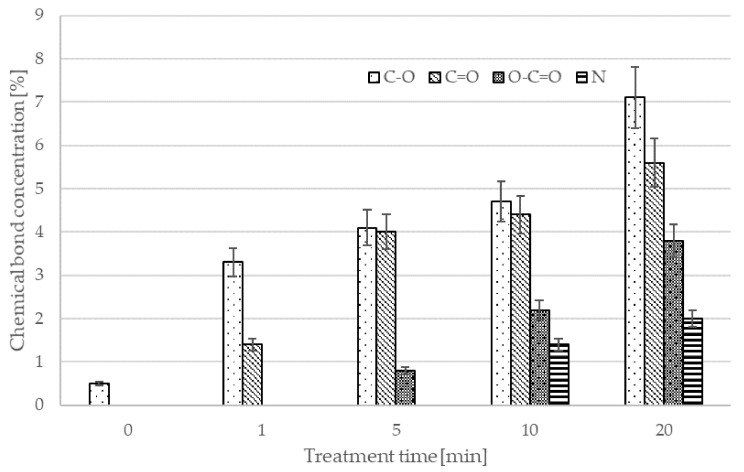
Dependence of chemical composition of the surface film on treatment time in air plasma.

The composition of the surface film as deduced from XPS survey spectra is shown in Figure 3. The pristine PE powder consists of carbon (and hydrogen, but H is not probed by XPS) with a small trace of oxygen. A 1 min treatment caused an increase of the oxygen concentration to 3.6 atomic%, and a 5 min treatment to 10.8 atomic%. The concentration of nitrogen remained below the detection limit of XPS, i.e., around 0.1 atomic%. Despite the fact that the wettability saturated after 5 min of treatment, the concentration of oxygen, as deduced from XPS, continued increasing with increasing treatment time and reached 15.6 atomic% and 2 atomic% nitrogen after 20-min treatment. The shear strength reached a peak in the 10 min treatment, with 12 atomic% oxygen and 1.4 atomic% nitrogen.

The prolonged treatment also caused the appearance of nitrogen in the surface film. Although the concentration remained up to about two atomic% even for the 20-min treatment, the value was well above the detection limit. The rather late appearance of nitrogen on the polymer surface could be explained by preferential oxidation of the polyolefin. Namely, the chemical affinity of reactive oxygen species (especially atoms and positively charged oxygen ions) was higher than the affinity of reactive nitrogen species [23]. Furthermore, the concentration of oxygen atoms in weakly ionized highly dissociated air plasma was orders of magnitude larger than the concentration of N atoms [20]. In any case, the wettability was saturated before a measurable amount of nitrogen appeared on the surface of the air-plasma-treated PE powder, as revealed by comparing Figure 2 and Figure 3.

**Figure 4:**
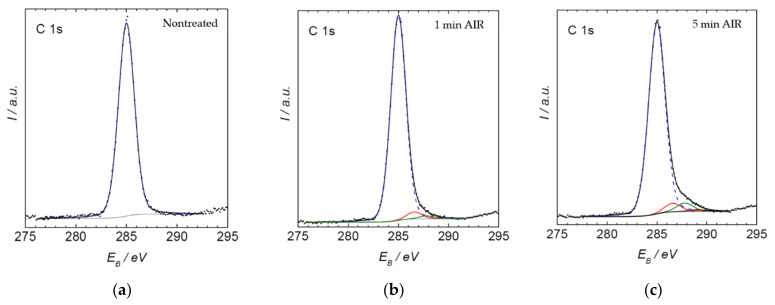

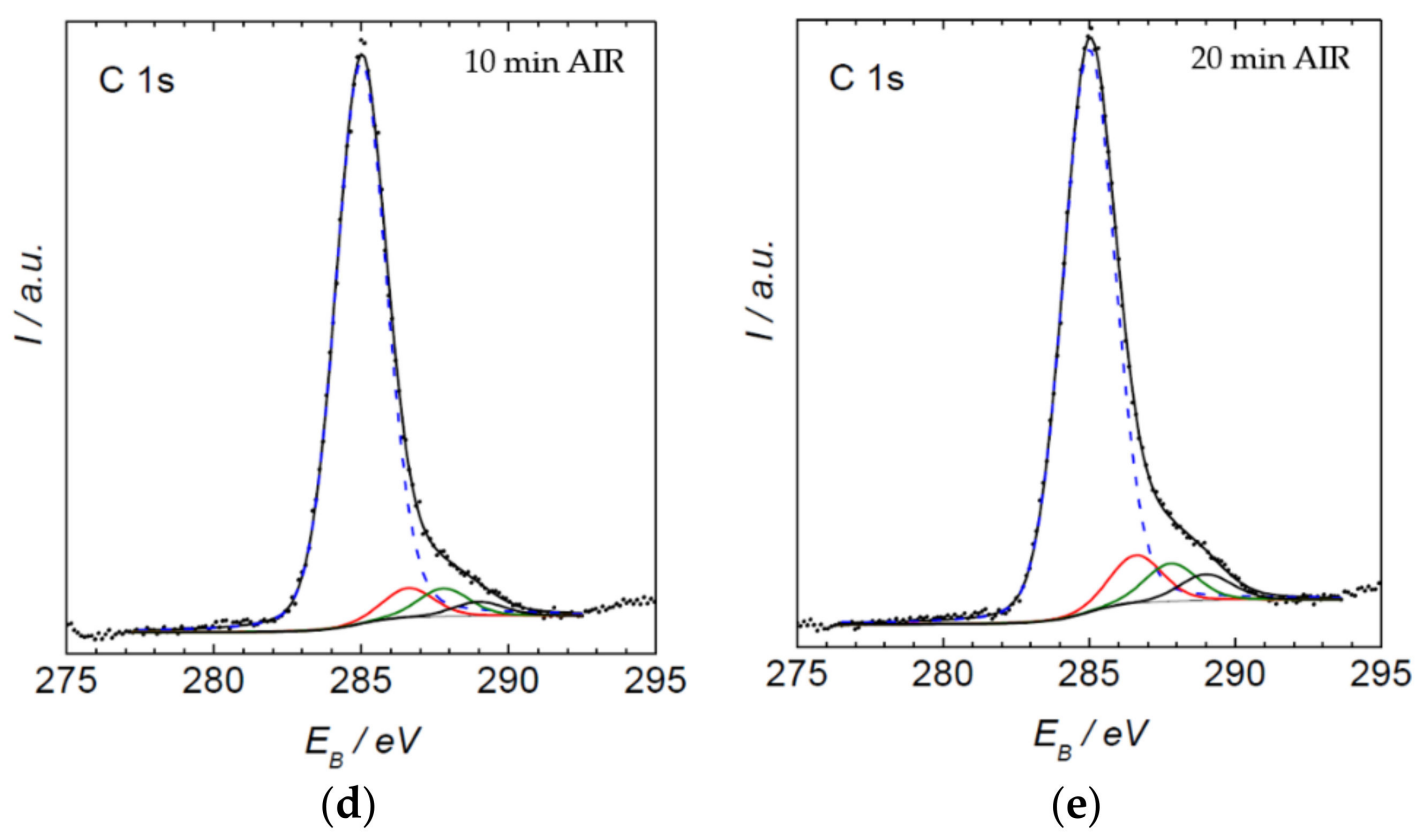
C1s spectrum of PE powder (**a**) non-treated and plasma-treated in air plasma for (**b**) 1 min, (**c**) 5 min, (**d**) 10 min and (**e**) 20 min. Blue line C-C bond, red line C-O bond, green line C=O bond, black line O-C=O bond.

The XPS survey spectra provide information on the composition of the surface film, revealing the surface chemistry. The evolution of the specific oxygen-containing functional groups on the polymer surface treated by air plasma was deduced from high-resolution XPS C1s spectra. The high-resolution spectrum for the air plasma treatment is shown in Figure 4. One can observe an asymmetric peak, typical for polyolefins with surface functional groups. The high-energy tail of the peak was deconvoluted, and the concentration of various functional groups was estimated from the area of each sub-peak. The evolution of the surface functional groups versus air-plasma treatment time is shown in Figure 4. The concentration of all functional groups increases with increasing treatment time, but different functionalities behave differently. For example, the concentration of O-C=O group on samples treated for 5 min was hardly measurable, whereas the concentration of C-O and C=O groups had already reached about 4%. Such a preferential functionalization with C-O and C=O groups has been already reported for polystyrene [24] and polyethylene [10,25,26] and is explained by step-wise reactions. First, one oxygen atom interacts chemically with the surface of a polyolefin to induce a hydroxyl group, and upon prolonged treatment, with O-atoms arriving onto the surface from the gas phase, functional groups richer in oxygen are formed. Unfortunately, polyolefins are not among the best materials for studying the kinetics of functional groups since the peak asymmetry, as shown in Figure 4, does not allow for interpretation with absolute certainty. However, they have a very significant uses throughout various industrial applications and therefore it is important to study them to the extent that is technically possible.

The discrepancy between the wettability as determined using the Washburn method and surface composition as deduced from XPS survey spectra for long treatment times was already observed by other authors. The brief literature survey presented in the Introduction shows little correlation between the surface functionalities and wettability. The discrepancy could be explained by the fact that the wettability is saturated already at a moderate concentration of oxygen in the surface film as deduced by XPS. Namely, the XPS probes polymer films with a thickness of several nanometers, whereas wettability reflects the surface itself. This discrepancy led us to conduct further experiments to examine the effect of the concentration of functional oxygen groups on the wettability and adhesion of plasma-treated powder. The contribution of the residual atmosphere (leakage) of semi-industrial equipment was used to control the amount of oxygen bonded in the functional surface groups. The contribution of residual atmosphere made up about 10% of the amount of used working gas as described above. This allowed us to correlate the amount of bonded oxygen with the effect of different working gases.

**Figure 5:**
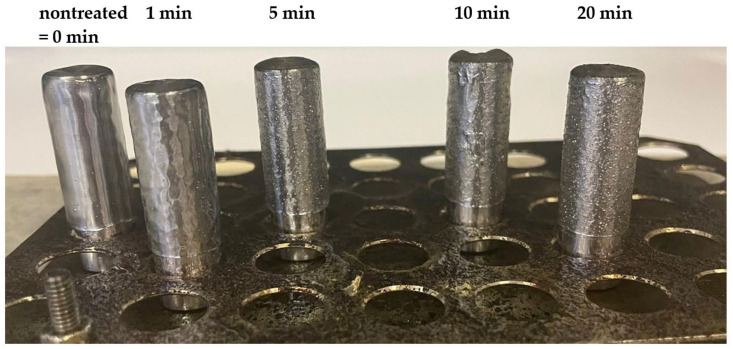
The process of melting polymer powder treated by air (timeline) into metal rod and surface smoothness.

**Figure 6:**
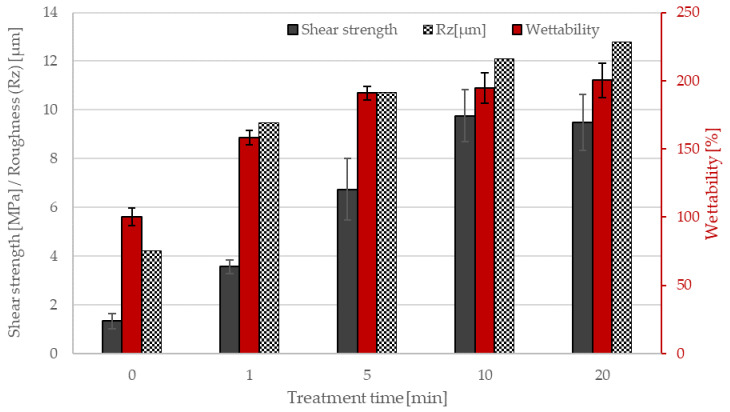
Comparation of surface roughness (Rz) with shear strength and wettability for air plasma timeline.

An etching effect of plasma, which results in increased surface roughness and thus contributes to increased wetting, is mainly attributed to argon or nitrogen, but also to hydrogen, ammonium or a mixture of hydrogen and nitrogen. In some cases, the used gases also showed a crosslinking effect that was prevented or combined with etching. However, different authors have observed different potencies for these gases, depending on the substrate, plasma source and its specifications, and the equipment used [2,27,28,29,30,31,32]. An increase in the roughness of sintered surfaces and the need for longer sintering times with longer plasma treatment times was observed on the timeline of air, as well as for the timeline of oxygen, in our last study [21]. This behavior was associated with an increase in the cross-linking effect with longer plasma treatment times. Thus, the formed shell of the cross-linked polymer is likely to prevent rapid and sufficient melting of the polymer powder on the metal rod during the adhesion test. The process of the sintering of the powder (treated in air plasma for different times) on a metal rod and the layer smoothness is shown in the Figure 5. A decreasing ability to melt and sinter in a uniform layer with increasing treatment time is clearly visible here. Figure 6 shows the dependence of surface roughness (Rz) with the length of plasma treatment in the air. We can see here that the 1 min plasma treatment (4 µm) already increased the surface roughness compared to the nontreated (9 µm) sample by more than two times. Nontreated powder shows a homogeneous, smooth surface of the sintered polymer layer. The surface roughness of the layer created from powder treated for 5 min is less uniform. The surface is becoming coarser, Rz is almost 11 µm, the imperfectly stretched particles of powder have failed to cover the entire surface, and tiny air bubbles are visible. We suppose that the crosslinked particle surface of the treated powder retards the sintering of particles. As we can see, the Figure 5. layer retains traces of the powder granularity, forming an “orange peel” surface structure and prevents the air bubbles from escaping from the material during the sintering process. The roughness rises with the length of the air plasma treatment, as well as the shear strength and wettability. The growth continued slightly after 10 min, and the highest value was observed for 20 min treatment, at almost 13 µm. The layer sintered using powder treated for 10 min had a roughness of 12 µm.

**Figure 7:**
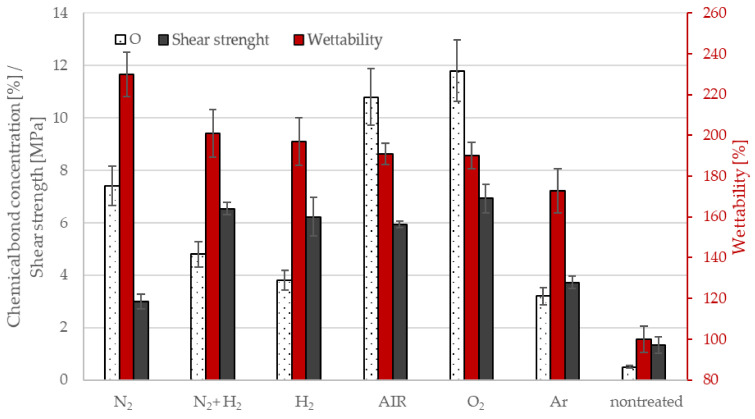
The composition of the surface film as deduced from XPS survey spectra for oxygen; wettability and shear strength in dependence on working atmosphere.

**Figure 8:**
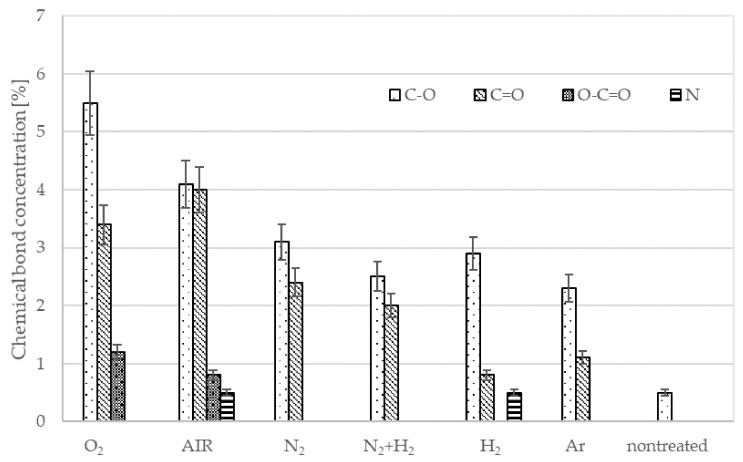
The composition of the surface film as deduced from XPS survey spectra.

The 5-min air plasma treatment is a breakthrough not only for wettability saturation, but also for industrial applicability. This treatment time corresponds in our experience to the borderline treatment time in the industrial production apparatus for this material. For this reason, we have used this time as a reference for experiments with other potentially usable technical gases in industry. We performed experiments with gases (oxygen, hydrogen, nitrogen, argon and a mixture (50/50 hydrogen with nitrogen)) for a 5 min treatment time. The values of wettability and shear strength, as well as the surface compositions and concentrations of detected functional groups, are shown in Figure 7 and Figure 8. As mentioned above, all samples were treated at the same conditions (i.e., discharge power about 1 kW, a pressure of 100 Pa, and the flow of gases of 100 sccm). The pump-down time was always three minutes, and the base pressure achieved after pumping for one minute was 12 Pa in all cases.

As seen in Figure 7, the wettability was significantly increased after plasma treatment for all samples. Differences in wettability between each treated sample largely fell within the brackets of experimental error, except for the results for N_2_ (230%), which achieved the highest wettability, and Ar (173%), which achieved the lowest wettability enhancement.

**Figure 9:**
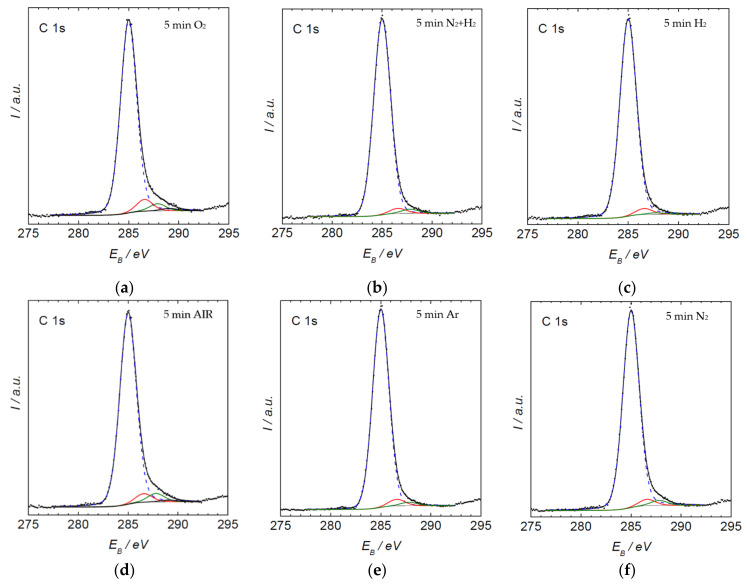
C1s spectrum of 5 min plasma-treated PE powder, treated in air plasma (**a**) oxygen, (**b**) nitrogen + hydrogen, (**c**) hydrogen, (**d**) air, (**e**) argon and (**f**) nitrogen. Blue line C-C bond, red line C-O bond, green line C=O bond, black line O-C=O bond.

Figure 8 allows the comparison of the content of new functional groups created through the plasma treatment for different gases. The surface functionalities differ depending on the type of gas. As expected, a rather large concentration of oxygen of about 11 atomic% was observed after treatment with plasmas containing oxygen, such as pure oxygen or air. Such treatments also enabled a rather large concentration of O-C=O functionalities of about 3–4%. Treatment with oxygen-free plasmas caused a lower, but definitely measurable O-concentration. This observation can be attributed to the rather large partial pressure of the residual atmosphere. As mentioned above, the vacuum level achieved after three minutes of pumping was moderate, at about 12 Pa. Such incomplete evacuation is typical for industrial reactors, and is useful for the treatment of polymers. The molecules comprising the residual atmosphere (water vapor in particular) dissociate due to plasma conditions, practically independently from the main gas. The flux of reactive species such as OH radicals and perhaps even O atoms is therefore large enough to cause oxidation of the polymer surface despite the large surface-to-mass ratio typical for polymer powder. The fluence achieved after the 5 min treatment, however, is too low to achieve a high oxygen concentration for cases when no oxygen-containing gas is introduced into the reactor intentionally. Therefore, it is necessary to expect that using different non-oxygen atmospheres at an industrial scale, such plasma-treated powders, will involve a significant amount of oxygen. All XPS C1 spectra are shown in Figure 9.

Despite the rather low oxygen concentration, the wettability of all samples, as shown in Figure 7, was much higher than that of the nontreated sample. This observation may be explained by taking into account other reactions, aside from functionalization with oxygen, that may contribute to increased wettability. As mentioned earlier, low-pressure non-equilibrium gaseous plasma is a source of UV and VUV radiation. For example, hydrogen plasma is an extremely rich source of radiation in the range of wavelengths from about 117 and 280 nm [33]. The radiation arises from transitions of highly excited neutral hydrogen molecules to the ground state. The radiation is not as extensive in the case of plasma sustained in nitrogen or a mixture of nitrogen and hydrogen, and the range of wavelengths is different, but according to Fanz et al. [33], the total intensity is of the same order of magnitude. The source of VUV radiation in the case of nitrogen is the Lyman–Birge–Hopfield series. Argon plasma is also a source of VUV radiation (but not UV) and is rich in Ar metastables that are capable of transferring their energy to molecules of the residual atmosphere. As the excitation energy of Ar metastables is higher than the dissociation energy of practically any molecule, the dissociation fraction of the residual gas is high. This effect may explain the measurable oxygen concentration and moderate wettability of polymer powder after treatment with argon plasma.

**Figure 10:**
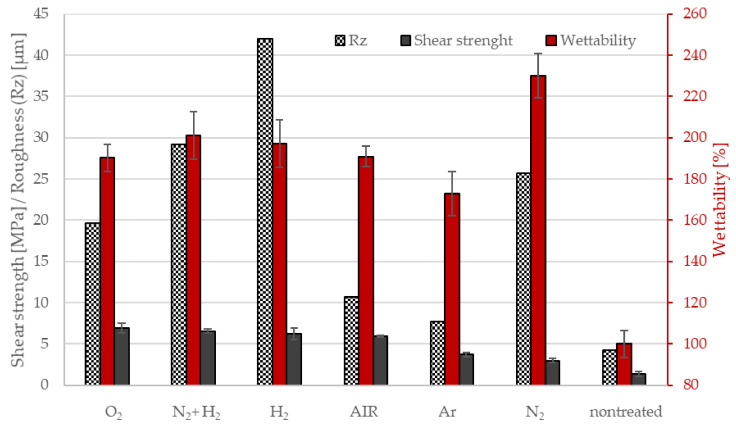
Comparison of surface roughness (Rz), shear strength and wettability for different gases.

**Figure 11:**
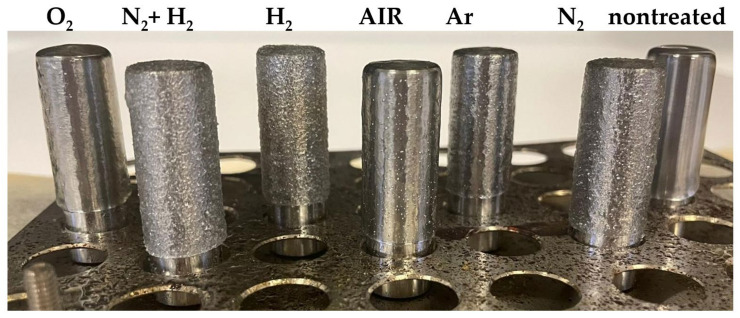
The process of melting polymer powder treated using different gases into metal rod and surface smoothness.

Air and oxygen gases are the most commonly used gases in the industrial plasma treatment of polymer powders [15] to bind functional groups that increase wetting but also result in adhesion to other materials. Using other gases in industrial applications to increase adhesion does not produce results better than using oxygen or standard air plasma, as seen in Figure 10, where values are sorted from the largest to the smallest shear strength: O_2_ ≥ N_2_ + H_2_ ≈ H_2_ ≈ AIR > Ar > N_2_ >> nontreated. Measurements of wettability, which are not too expensive or time-consuming, are commonly used for quantifications in industry. However, the measured values show that using wettability to estimate the adhesion of plasma-treated powders in different atmosphere (not containing oxygen) can result in misleading conclusions due to increased wetting, which does not bring about increased strength, as the wettability and adhesion values for the nitrogen atmosphere have shown. Here, increased wettability (the highest of the tested atmospheres, 230%) is likely to be supported by morphological changes on the surface (roughness, together with the crosslinking effect, as illustrated well in Figure 11). These surface changes, however, limited the formation of metal adhesion in the powder treated in this atmosphere. This is despite the higher content of bonded functional groups (nitrogen at the detection limit of 0.5 atomic% and oxygen at 7.3 atomic%, corresponding to the third-highest oxygen content after oxygen (11.8 atomic%) and air (10.8 atomic%) atmosphere).

**Figure 12:**
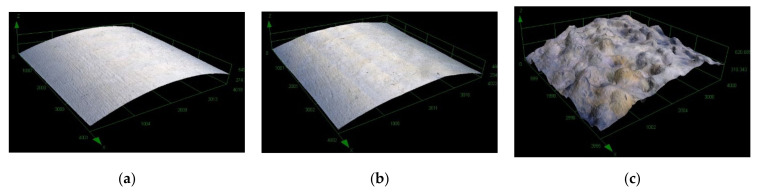
Profiles of metal rods with sintered powder: (**a**) nontreated and treated (**b**) for 5 min with air and (**c**) for 5 min with hydrogen.

Surface roughness (Rz) was also determined for these samples (Figure 10). The roughness trend was H_2_ > N_2_ + H_2_ > N_2_ > O_2_> AIR > Ar > nontreated. Figure 12 shows examples of surfaces sintered with powder. The smoothest was the nontreated sample, a rougher sample was treated for 5 min in air and the roughest was treated for 5 min in hydrogen plasma.

From an industrial perspective, it might be interesting to use a working gas combining nitrogen and hydrogen or hydrogen alone, offering a strong increase in wettability (201% and 197%), as well as a high shear strength (6.5 MPa and 6.2 MPa), despite the relatively low oxygen content on the surface (4.8 atomic% and 3.8 atomic%). These results are especially interesting because of the increase in wettability and strength without the need to load the surface with high oxidation, which occurs when oxygen or air is used in the working atmosphere. Comparing the hydrogen and argon atmospheres, similar representations of functional groups were found on the surface. After the argon treatment, the powder surface contained nitrogen at the detection limit of 0.5 atomic% and oxygen at 3.2 atomic%. After the hydrogen-treatment the powder surface contained oxygen at 3.8 atomic%. By comparing the hydrogen and argon atmospheres from the wettability perspective (increases to 197% and 172%, respectively), it can be assumed that there was a smaller increase in roughness on the surface of the polymer in the argon atmosphere than in the hydrogen atmosphere. Regarding the shear strength, it should be pointed out that argon treatment (3.7 MPa) reached almost half the strength of hydrogen treatment (6.2 MPa). This disparity could be explained by more intense crosslinking caused by the argon treatment. However, when comparing the roughness of the sintered layer on a metal rod, we observed increased roughness of the layer surface for the hydrogen treatment, but not for the argon treatment. The only major difference in the discrepancy between the strength and content of the functional surface groups was observed for nitrogen at the detection limit of 0.5 atomic % on the surface. This phenomenon was seen in nitrogen treatment, where despite high wettability and high oxygen content (7.4 atomic%), very low adhesion (3 MPa) was measured. It seems that the nitrogen content reduced the shear strength. Similar behavior was observed for the treatment time series with air, where the strength reached its maximum at the first detection of bonded nitrogen (10 min). With a longer treatment time, the strength did not increase despite the increase in the oxygen concentration (from 12 to 15.6 atomic%). The element that could thus negatively affect the strength might just be the increase of nitrogen on a surface (from 1.4 to 2 atomic%). The theory of a negative influence of nitrogen is also supported by the measurements previously made by our team, where the influence of oxygen treatment length on wettability and adhesion [21] was investigated. Nitrogen was not detected in this oxygen plasma treatment time series and the adhesion increased with treatment time even for 20 min, which was the highest treatment time tested. In the air plasma time series, adhesion stopped increasing with the first nitrogen detected on the surface (10 min). With longer treatment times, adhesion did not increase. Using hydrogen in the atmosphere, it seems possible to limit the amount of bonded nitrogen on the surface, as suggested by the mixed nitrogen-hydrogen atmosphere. The possibility of adding a small amount of hydrogen to an air atmosphere could thus potentially eliminate the bonding of nitrogen to the surface, limiting its potentially negative effect on adhesion.

## 4. Conclusions

Plasma treatment of polymer powder (LLDPE) was carried our using semi-industrial equipment. In the first step, a timeline in the air atmosphere was prepared. With the longest treatment time of 20 min, we achieved a saturation of wettability and adhesion. The wettability reached 200% and the adhesion increased by more than seven times—from 1.3 to 9.5 MPa. The concentration of surface oxygen increased from 0.5% to 15.6%, and surface roughness (Rz) increased from 4 to almost 13 µm. The treatment time of 5 min was found to be satisfactory for possible industrial applications. This duration was selected for the use of other technical gases (oxygen, hydrogen, nitrogen, argon and a mixture (50/50 hydrogen with nitrogen)) for the plasma treatment of the powder. Nitrogen reached the highest wettability with an increase of 230%; the highest adhesion of almost 7 MPa was shown by the oxygen atmosphere, together with the highest content of oxygen-containing groups (almost 12%) on the surface. The roughest surface was observed for hydrogen treatment (Rz = 42 µm) due to surface etching by hydrogen radicals. The smallest increase in adhesion was observed for argon and nitrogen treatments; only these two atmospheres contained detectable concentrations of nitrogen on the surface of the plasma-treated powder (0.5%). These values confirm the suitability of using standard oxygen or a cheaper air atmosphere for the purpose of increasing the adhesion of polymer powders. In contrast, it seems interesting to use the nitrogen atmosphere to increase the wettability of the powders. Furthermore, the use of a hydrogen atmosphere is interesting due to increased roughness of the surface after sintering, e.g., in multi-layer composites, where mechanical anchorage plays a role.
